# Medical school accreditation in Australia: Issues involved in assessing major changes and new programs

**DOI:** 10.3352/jeehp.2011.8.6

**Published:** 2011-06-08

**Authors:** Michael J. Field

**Affiliations:** Sydney Medical School, The University of Sydney, Sydney, NSW, Australia.

**Keywords:** Accreditation, Medical school, Australia, Educational program

## Abstract

The Australian Medical Council (AMC) is an independent company for quality assurance and quality improvement in medical education in Australia and New Zealand. Accreditation procedures for the 20 medical schools in these two countries are somewhat different for three different circumstances or stages of school development: existing medical schools, established courses undergoing major changes, and new schools. This paper will outline some issues involved in major changes to existing courses, and new medical school programs. Major changes have included change from a 6 year undergraduate course to a 5 year undergraduate course or 4 year graduate-entry course, introduction of a lateral graduate-entry stream, new domestic site of course delivery, offshore course delivery, joint program between two universities, and major change to curriculum. In the case of a major change assessment, accreditation of the new or revised course may be granted for a period up to two years after the full course has been implemented. In the assessment of proposals for introduction of new medical courses, six issues needing careful consideration have arisen: forward planning, academic staffing, adequate clinical experience, acceptable research program, adequacy of resources, postgraduate training program and employment.

## INTRODUCTION

The Australian Medical Council is an independent company with responsibility under the law for quality assurance and quality improvement in medical education, at both undergraduate and postgraduate vocational levels, in Australia and New Zealand [1]. In the case of basic (undergraduate) medical education, accreditation of medical school programs by the AMC leads to eligibility of graduates from those schools for registration as a medical practitioner in Australia and New Zealand, following a one-year period of supervised practice as an intern following graduation.

The overall goal of basic medical education is defined by the AMC as follows: "to develop junior doctors who possess attributes that will ensure that they are competent to practise safely and effectively as interns in Australia or New Zealand, and that they have an appropriate foundation for lifelong learning and for further training in any branch of medicine."

In accrediting programs of basic medical education, the AMC lists 40 attributes required of graduates from medical school, and sets standards for medical schools and medical courses which result in the award of a primary medical degree [2]. Of the required attributes, 12 are in the domain of knowledge and understanding, 13 are skills, and 15 relate to attitudes as they affect professional behaviour. The standards for accreditation are consistent with those recommended by the World Federation for Medical Education, and fall under the following eight headings:

1. Context of the medical school (including governance)
2. Outcomes of the medical course (including mission)
3. The medical curriculum
4. Teaching and learning
5. Assessment of student learning
6. Monitoring and evaluation
7. Students (including admission and support)
8. Educational resources

Accreditation of medical schools against these standards is performed according to well-defined procedures [2] in three stages:

- A written submission involving a rigorous self-assessment is prepared by the school,
- A site visit is conducted by an AMC-appointed a team of peer reviewers, and
- A written report is prepared containing a recommendation on the outcome of accreditation, which is ultimately decided by the AMC directors.

These procedures are somewhat different for schools or courses undergoing accreditation in three different circumstances or stages of development: existing medical schools, established courses undergoing major changes, and new schools. A summary of outcomes of AMC accreditation activity against these three categories is given in Table 1.

This paper will outline some issues involved in assessing submissions in the latter two categories, that is, major changes to existing courses, and new medical school programs.

## MAJOR CHANGES

Medical education in Australia and New Zealand has undergone a period of renewal over the past two decades, and this has involved many established schools submitting proposals to make major changes to their programs, involving particularly changes in student admission procedures and curriculum.

The AMC defines a "major change" as

- A change in the length or format of the course,
- A substantial change in educational philosophy, objectives, emphasis or institutional setting,
- A substantial change in student numbers relative to resources, or
- Changes forced by a major reduction in resources.

Using these criteria, there have been a total of 18 major change assessments conducted over the past decade and a half. The basis of these changes is given in Table 2 (in some cases more than one major change was assessed at the same time).

The possible outcomes of a major change assessment are different from those following a re-accreditation of an established medical school. In the latter case, the maximum outcome is 10 years' accreditation, administered as an initial six year period with the potential for a four-year extension following the submission of a satisfactory comprehensive report in year five. In the case of a major change assessment, accreditation of the new or revised course may be granted for a period up to two years after the full course has been implemented, subject to any conditions being addressed within a specific period of time, and this is extendable by up to four years following submission of a satisfactory comprehensive progress report. The other major option is refusal of accreditation where the school has not satisfied the AMC that the complete medical course can be implemented at a level consistent with AMC accreditation standards.

One example may serve to illustrate the challenges which arise in major change proposals. Since 2006, two established schools have put forward plans to deliver the medical course, in part or in full, in an overseas location. To provide guidance in this situation, the AMC prepared a document giving the requirements which need to be satisfied for such proposals to be considered, and the specific issues which need to be addressed for accreditation of the offshore program to be successful. Chief among these were the requirement for direct involvement by the academic staff of the parent medical school in the development, governance and delivery of the overseas course, and the need to demonstrate equivalence to Australian education and clinical experience in the overseas location. While it was not easy for the schools to meet these criteria in their initial submissions, in both cases sufficient information and evidence were ultimately provided to allow accreditation of the major changes to be approved.

## NEW MEDICAL SCHOOLS

In the past decade, there has been a dramatic expansion in medical school capacity and the output of graduates in Australia [3], responding to the realisation that the existing medical workforce was inadequate in relation to community growth and increasing demand. A particular problem was the maldistribution of doctors and other healthcare workers throughout Australia, the central metropolitan areas of the major capital cities being relatively well provided, while outer metropolitan and rural and remote locations were seriously undersupplied with medical workers. The Australian Government took action in three ways to address this need: student places in existing schools were increased, new schools were established in underserved areas, and rural clinical schools were set up in key centres of rural population.

The scale of this increase is shown by the data in Table 3. By 2012, there will have been more than a doubling of the output of graduates from Australian medical schools compared to that in the year 2000. These figures include a subset of students recruited internationally, which comprise around 20% of the overall medical student population, and relatively few of these are expected to stay in Australia for their practising career. It should also be mentioned that another 500 to 1,000 medical graduates of overseas medical schools are admitted to practice in Australia each year, following a rigorous process of assessment also conducted by the AMC.

A number of common issues have arisen in the process of assessing the new schools for accreditation against AMC standards. Six areas are worth special comment.

The first issue relates to realistic forward planning. Of the nine new medical schools established in Australia since 1999, five did not provide a sufficiently well developed first stage accreditation submission to convince the AMC that the institution would be able to implement a medical program. Five were required to submit multiple proposals before they could proceed to accreditation. Four of these have had subsequent difficulties in meeting AMC accreditation standards, necessitating significant additional work by and cost to the school once established. Common concerns about planning processes have related to lack of understanding of the requirements for a successful medical school, key appointments being delayed, and lack of planning for clinical placements in a wide range of health care settings, and poor communication of course requirements to clinicians who are expected to supervise and assess medical students. Similar problems have occurred when established schools fail to plan for major change.

Second, academic staffing has been a challenge for all schools, in that there is limited availability of appropriate academic staff in all relevant fields in Australia, and inevitably some core disciplines have been relatively underserved. A particular challenge for new schools is the recruitment of leaders in curriculum design and implementation, and in a number of cases this has required hiring qualified educators from overseas. An initial concern was the possibility that recruitment from existing schools would lead to weakening of those faculties, but this has not proven to be a major issue to date.

Next, providing students with adequate clinical experience has also required some innovative approaches [4]. With the support of several government programs, a wider range of settings for clinical placements has been developed, though this has inevitably involved some overlap of student allocations between existing schools and new schools, in both hospital and community environments. The AMC has assisted in managing these situations by setting out requirements for joint planning, including the need for having clear agreements on sharing clinical resources. At the same time, opportunities for collaboration between different schools have arisen in the delivery of equivalent clinical experience to their students.

A fourth area of challenge has been the requirement for schools to demonstrate an adequate research base to underpin their education programs. Naturally new schools require time to set up suitable research programs, but the AMC has been prepared to accept interim markers of progress in this direction, such as evidence for development of suitable staffing and structures for future research activity.

New schools are also required to demonstrate adequacy of resources, both financial and physical, to implement and sustain the medical course. Clearly there is a potential risk to the viability of small schools, and assurances are needed from the parent University and the relevant Health Service that sufficient resources will be made available for the school to succeed.

Finally, the rapid expansion of the medical student population has put pressure on early postgraduate clinical training programs and employment opportunities for all the new graduates. This challenge is gradually being addressed at the level of state and national governments, where joint planning with postgraduate medical councils and health authorities has been required to ensure the availability of sufficient quality intern places for the increasing number of graduates. Ongoing attention will be needed to manage the flow-on implications for supervision of trainees seeking to enter the colleges responsible for specialist training in the decades to come.

## CONCLUSION

The Australian Medical Council's accreditation system has been successfully modified for medical schools undertaking major changes and for new medical schools. Of the major changes assessed recently, the operation of a school in an overseas location has been an example of a challenging proposal needing careful evaluation, leading ultimately to accreditation. In the assessment of proposals for establishing new medical schools, six common issues have arisen: forward planning; academic staffing; adequate clinical experience; acceptable research program; adequacy of resources; postgraduate training program and employment.

## Figures and Tables

**Table 1:**
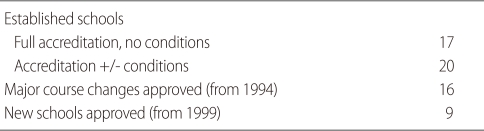
Medical school accreditation outcomes since 1989

**Table 2:**
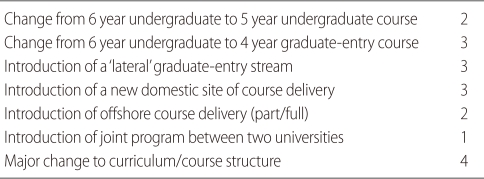
Major change proposals by Australian medical schools

**Table 3:**
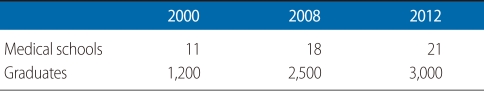
Growth in Australian medical schools
